# An Exploratory Study on the Pathways of Cr (VI) Reduction in Sulfate-reducing Up-flow Anaerobic Sludge Bed (UASB) Reactor

**DOI:** 10.1038/srep23694

**Published:** 2016-03-29

**Authors:** Jin Qian, Li Wei, Rulong Liu, Feng Jiang, Xiaodi Hao, Guang-Hao Chen

**Affiliations:** 1 School of Natural and Applied Sciences, Northwestern Polytechnical University, Xi’an, China; 2 Department of Civil and Environmental Engineering, The Hong Kong University of Science and Technology, Clear Water Bay, Kowloon, Hong Kong, China; 3 Water Technology Center, The Hong Kong University of Science and Technology, Hong Kong, China; 4 Hong Kong Branch of Chinese National Engineering Research Center for Control & Treatment of Heavy Metal Pollution, The Hong Kong University of Science and Technology, Hong Kong, China; 5 Wastewater Treatment Laboratory, FYT Graduate School, The Hong Kong University of Science and Technology, Nansha, Guangzhou, China; 6 School of Chemistry & Environment, South China Normal University, Guangzhou, China; 7 Beijing University of Civil Engineering and Architecture, Beijing, China

## Abstract

Electroplating wastewater contains both Cr (VI) and sulfate. So Cr (VI) removal under sulfate-rich condition is quite complicated. This study mainly investigates the pathways for Cr (VI) removal under biological sulfate-reducing condition in the up-flow anaerobic sludge bed (UASB) reactor. Two potential pathways are found for the removal of Cr (VI). The first one is the sulfidogenesis-induced Cr (VI) reduction pathway (for 90% Cr (VI) removal), in which Cr (VI) is reduced by sulfide generated from biological reduction of sulfate. The second one leads to direct reduction of Cr (VI) which is utilized by bacteria as the electron acceptor (for 10% Cr (VI) removal). Batch test results confirmed that sulfide was oxidized to elemental sulfur instead of sulfate during Cr (VI) reduction. The produced extracellular polymeric substances (EPS) provided protection to the microbes, resulting in effective removal of Cr (VI). Sulfate-reducing bacteria (SRB) genera accounted for 11.1% of the total bacterial community; thus they could be the major organisms mediating the sulfidogenesis-induced reduction of Cr (VI). In addition, chromate-utilizing genera (e.g. *Microbacterium*) were also detected, which were possibly responsible for the direct reduction of Cr (VI) using organics as the electron donor and Cr (VI) as the electron acceptor.

Industrial wastewater from electroplating, leather tanning, wood preservation, mine tailings, etc. are responsible for discharge of chromium, with oxidation states ranging from −2 to +6, in to the environment^1^. In nature, Chromium primarily exists in hexavalent state (Cr (VI)) and/or trivalent state (Cr (III)), both of which are ecologically important with respect to their environmental stability^2^. Cr (VI) (mainly CrO_4_^2−^ at neutral pH or alkaline conditions) is either carcinogenic or mutagenic to living microorganisms^3^. Therefore, several governments such as the Environmental Protection Agency (EPA) in the USA and the European Union (EU) have mandated a stringent discharge limit of 0.05 mg Cr (VI)/L to protect surface waters from contamination of Cr (VI)^4^,^5^. In contrast, Cr (III), mainly in the form of Cr(OH)_3_ at neutral pH, is 100 times less toxic and 1000 times less mutagenic than Cr (VI) because of its low solubility^6^,^7^,^8^.

Besides chromium, electroplating wastewater (EW) contains large amount of sulfate as sulfuric acid is applied to polish the surface of metals. The presence of sulfate makes the detoxification process more complicated and expensive. Based on the previous studies, Cr (VI) can be removed chemically by reducing agents such as Fe^2+ ^7,9 or biologically via microbial conversion of Cr (VI) to Cr (III)^10^,^11^. The chemical removal process consumes large amounts of chemicals^12^. In comparison to this, biological conversion is attractive and cost-effective because of its low chemical requirement, low energy consumption, and increased removal efficiency^13^,^14^. When the sulfate content is high in these wastewaters, sulfidogenesis prevails under anaerobic condition in which sulfate-reducing bacteria (SRB) reduce sulfate to sulfide using organics as electron donors. Sulfide has been reported to be able to reduce Cr (VI) to Cr (III)^15^,^16^. Thus, sulfidogenesis is a promising way to facilitate simultaneous removal of organics and Cr (VI), as described by Eqs 1, 2, 3 17,18,19.


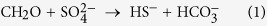



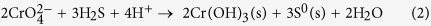



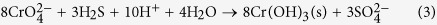


Thermodynamically, Cr (VI) can also be directly reduced to compounds of insoluble Cr (III) under anaerobic condition by *Desulfovibrio*-like species affiliated to the SRB group and chromate-reducing bacteria^20^,^21^,^22^,^23^. Since CrO_4_^2−^ has a structure similar to SO_4_^2−^ (both having four tetrahedrally arranged oxygen atoms and two negative charges), it may possess similar bioactivity to SO_4_^2−^ 24,25,26. Under specific conditions, SRB and other chromate-reducing microbial species can reduce Cr (VI) to Cr (III) directly through their cytochromes, as described by Eq. 4 27. Therefore, for industrial wastewaters, such as electroplating wastewater, which contain Cr (VI), sulfate and organics, it is possible to simultaneously remove Cr (VI) and organics through either sulfidogenic activity (biological sulfate reducing to sulfide and sulfide reducing Cr^6+^ to Cr^3+^) or direct Cr^6+^ reduction (with Cr (VI) and organics as electron acceptor and donor respectively) in an anaerobic reactor.


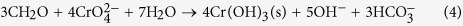


Durai and Rajasimman *et al.*^28^ reviewed the Cr (VI) removal performances with different types of reactor in different studies and concluded that UASB reactors can achieve more efficient removal of Cr (VI) than conventional reactors, such as aerobic membrane bioreactors or sequential batch reactors. Moreover, our research team carried out a study on Hg and Pb removals from wet flue desulfurization (WFGD) wastewater in a UASB reactor for 50 days, and the results showed that the removal efficiencies of both metals were above 99.5% when the metal loading rates were 9.2 g Pb (II)/d/m^3^ and 2.6 Hg (II)/d/m^3^, respectively (data will be reported soon). Therefore, in this study we focused on the UASB reactor for Cr (VI) removal.

However, the potential for biological removal capacity of Cr (VI) is limited by low SRB growth under high Cr (VI) concentration^29^ because SRB may be more sensitive to Cr (VI) than other anaerobic bacteria^23^,^30^. Marsh *et al.*^31^ reported that sulfate-reducing activity was totally inhibited when the Cr (VI) concentration was higher than 25 mg/L. Therefore, the feasibility of the biological removal of Cr (VI) at high concentration in the UASB reactor needs to be assessed. Besides, in order to increase the potential for removal of Cr (VI) through bioactivity, it is necessary to understand detailed pathways of Cr (VI) reduction for the contribution of Cr (VI) removal in the UASB reactor, which has not yet been explored though the sulfidogenesis-induced Cr (VI) reduction and direct Cr (VI) reduction were reported in the literature. Morevoer, most of the previous studies on biological removal are mainly focused on the pure instead of the mixed sludge. Hence, this study mainly focuses on: 1) the feasibility and performance of the biological removal of Cr (VI) in EW at high concentration by the anaerobic sludge in the UASB reactor and 2) the pathways for Cr (VI) reduction in the UASB reactor. Batch tests with the reactor’s sludge were also conducted under different pH conditions to determine the biomass-specific removal rate of Cr (VI), with different control reactors set-up to find the pathways of Cr (VI) reduction in the UASB reactor. Microbial community analysis of the sludge was subsequently carried out to further explore a possible mechanism for the removal of Cr (VI) in the UASB reactor. The findings of this study will shed light on the advancement of Cr (VI) removal in the UASB reactor.

## Results

### Removal of Cr (VI) in UASB reactor

**Figure 1:**
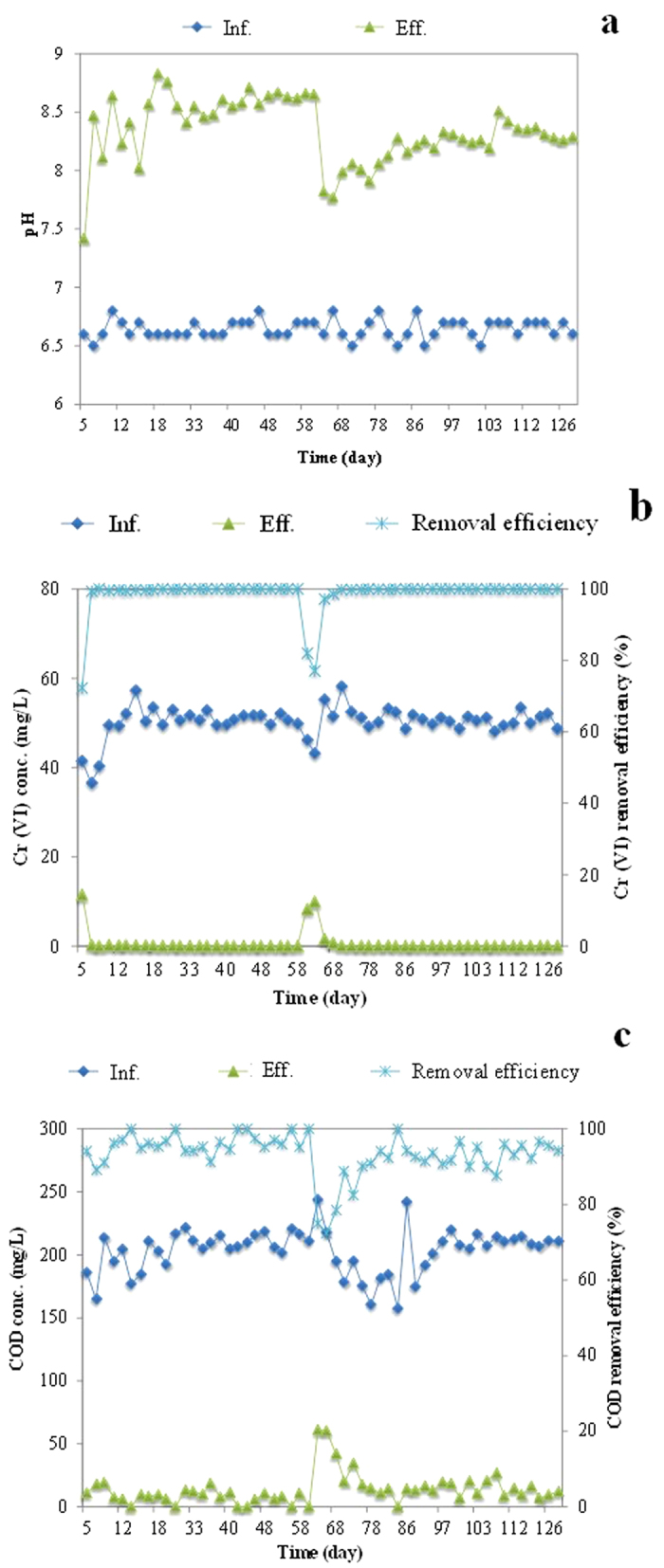
Profile of pH (**a**) Cr (VI) removal (**b**) and COD removal (**c**) in UASB reactor.

The influent pH ranged 6.5–6.8 while the effluent pH ranged 7.7–8.5; thus there was an increase in pH, in both Phase 1 and Phase 2 (Fig. 1a). This was mainly due to increased alkalinity and reduced acidity as described by Eqs 1, 2, 3, 4. In the pH range of 6.5–8.5 through the UASB reactor, Cr (VI) mainly existed as CrO_4_^2−^ and HCrO_4_^−3^ while Cr (III) mainly existed as Cr(OH)_3_^32^.

Figure 1b shows the performance of the UASB reactor in terms of the removal of Cr (VI). Apparently, no Cr (VI) was detected in the effluent for most of the operating period, except at the beginning of Phase 2 when the HRT decreased from 24 to 12 hours. Such decrease in the Cr removal efficiency could be due to the changes in surrounding conditions during the transition period (particularly changes in HRT, from 24 to 12 h). The results indicate that Cr (VI) was totally removed in both phases 1 and 2 of the UASB reactor despite the influent Cr (VI) concentration being as high as 50 mg/L (Fig. 1b). As a result, Cr (VI) removal capacity was 7.7 mg Cr (VI)/g VSS/d. Consequently, the COD removal efficiency was more than 95% and 90% in Phase 1 and Phase 2, respectively (Fig. 1c). It suggests that such high concentration of Cr (VI) has no significant impact on bioactivity. In contrast, Klonowska *et al.*^23^ reported that sulfate-reducing activity was depressed by very low Cr (VI) concentration of 2.6 mg/L in the pure culture of SRB. The discrepancy in these observations can be explained by the fact that a different bacterial culture was used in the present study. The inhibition/toxicity of Cr (VI) appears to be more potent in pure cultures than in mixed cultures^33^. Lowe *et al.*^34^ also pointed that mixed bacterial cultures are more tolerant to Cr (VI) than isolates.

### EPS concentrations in the UASB reactor

**Figure 2:**
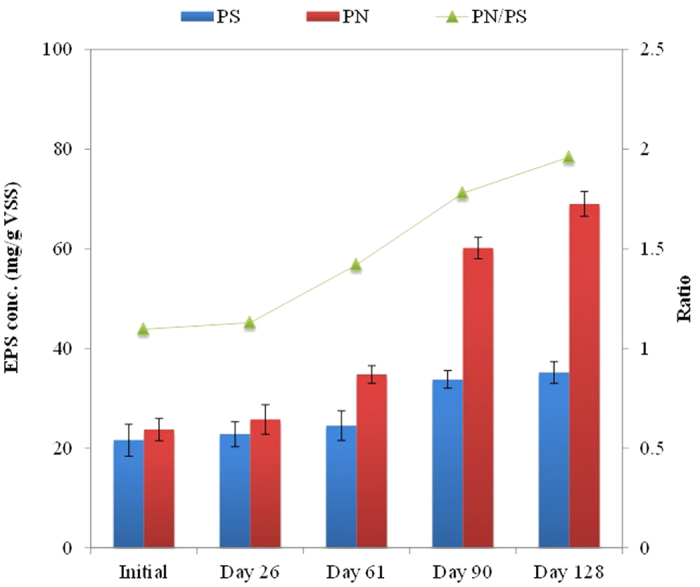
EPS (including both PN and PS) profile of UASB reactor.

Initially, the polysaccharides (PS) and protein (PN) of the pre-cultured sludge samples were 21.6 and 23.67 mg/g VSS, respectively. Both PS and PN of the sludge gradually increased to 24.5 and 34.8 mg/g VSS at the end of Phase 1 (Day 61) and continued to increase to 35.2 and 69 mg/g VSS respectively at the end of Phase 2 (Day 128) (Fig. 2). In addition, the ratio of PN to PS was found to gradually increase during the operations of the UASB reactor. Other studies also report the presence of toxic metals and chemicals which stimulated production of EPS, yielding more PN than PS^35^,^36^,^37^,^38^,^39^,^40^,^41^,^42^,^43^,^44^,^45^,^46^,^47^. EPS formed a protective shield for the microbial cells against adverse influences from the external environment^38^. Thus, the produced EPS may explain high biological activity in the UASB reactor even under a high Cr (VI) concentration of 50 mg/L as well as the effective removal of Cr (VI) in the reactor.

### Sludge production in UASB reactor

Sludge produced in the UASB reactor constitutes two sources. One is biological sludge produced from biomass growth and the other is chemical sludge produced from Cr precipitation and possible S^0^ sulfur generation in the reactor. The biological sludge production could be due to the accumulation of volatile suspended solids in the reactor (see Fig. S1 in SI). Biomass was calculated by taking the average of the volatile suspended solid (VSS) concentration of samples at the top, middle and the bottom of the sludge bed, with the sludge loss in periodically sampling and wash out taken into account. As shown in Fig. S1, the biological sludge production rate, i.e. accumulative specific biomass growth rate is 0.028 g VSS/g COD_removed_ (accumulative growth rate is 0.011 g VSS/d, average COD removal rate is 0.386 g COD/d; detailed calculation can be seen in Fig. S1a), which is very close to the value of 0.02–0.03 g VSS/g COD_removed_ in biological sulfate-reducing reactor^39^. Therefore, this is another evidence to prove that sulfate reduction-induced Cr (VI) removal is mainly responsible for removal of both organics and Cr (VI) in the UASB reactor. The chemical sludge production rate is about 0.397 g ISS/d (see Fig. S1a,b), which can be attributed to the accumulation of inorganic suspended solids (ISS, calculated as: ISS = TSS-VSS) in the reactor. The chemical sludge may include Cr precipitation and S^0^ production in the reactor. Following the findings of Cheung and Gu^32^, the Cr (influent concentration of 50 mg/L at HRT of 12h with 100% removal) should be precipitated as Cr(OH)_3_ under pH of 6.5–8.5 in our study. Therefore, the chemical sludge produced due to Cr precipitation should theoretically be 0.22 g Cr(OH)_3_/d. Also, as mentioned above, the sulfide (reduced from sulfate) was most possibly oxidized to S^0^ and the stoichiometric ratio of Cr to S^0^ is 0.92 (Eq. 2). Therefore, the chemical sludge production from S^0^ generation would be 0.1 g S^0^/d. As a result, chemical sludge production of 0.397 g/d can almost be recovered as Cr(OH)_3_ (0.22 g/d) precipitation and S^0^ generation (0.1 g/d). Based on the results of both biological and chemical sludge production, total sludge production rate is estimated to be 0.408 g TSS/d.

### Batch test: possible pathways for Cr (VI) reduction and effect of pH on Cr (VI) reduction

In this study, there are four possible processes leading to the removal of Cr (VI) in the UASB reactor, 1) chemical adsorption on to organic substrates, 2) biosorption on to microorganisms, 3) direct biological reduction of Cr (VI) to Cr (III) mediated by bacterial metabolism, and 4) indirect removal by sulfate reduction (sulfate to sulfide) coupled with abiotic reduction of Cr (VI) by sulfide.

Metals can be removed in bioreactors via weak adsorption onto organic substrates^40^. However, in this study no loss of Cr (VI) was found through adsorption (Fig. S2a) during the operation of the non-biomass reactors (Control Reactors 1 to 4; see Table 1). Besides biological reduction, biosorption was reported to be significant for efficient removal of Cr (VI) biologically in both pure cultures and mixed sludge^41^,^42^. The results in this study obtained for non-organics/sulfate reactors (Control Reactors 5 to 8, see Fig. S2b in SI) show that less than 3% Cr (VI) was removed in the absence of electron donors (organics). Therefore, adsorption by biomass cannot be a major mechanism contributing to the removal of Cr (VI) in the UASB reactor. Non-sulfate reactors (Control Reactors 9 to 12) demonstrated the effect of direct reduction of Cr (VI) in the UASB reactor (without sulfate), when Cr (VI) was the sole electron acceptor. Under such condition, 15–20% chromate was reduced at the end of the test (see Fig. S2c). This suggests that besides the sulfidogenesis-induced removal of Cr (VI), the direct reduction of Cr (VI) could also contribute to Cr (VI) removal in the UASB reactor.

Without dosing Cr (VI) in non-Cr (VI) reactors (Control Reactors 13 to 16), where the pH was in the range of 6.0–9.0, the activity of SRB in terms of biomass-specific sulfate reduction rate was higher in these reactors than in Batch Reactors 1 to 4 in which Cr (VI) was also dosed. It is noteworthy that these batch reactors were operated at the same pH range (6.0–9.0) as shown in Table 2. After dosing 50 mg/L Cr (VI) in each reactor, the sulfate-reducing activity in terms of biomass-specific sulfate reduction rate was inhibited by 21–47%. It can, therefore, be concluded that sulfate reducing activity was negatively affected to some extent by the presence of Cr (VI)^23^.

Among all the pH values tested, biomass-specific rates of Cr (VI) removal and sulfate reduction were the highest at pH 8.0 (see Table 2; detailed batch test results can be seen from Fig. S3). The highest sulfate-reducing rate in Batch Reactor 3 corresponds to the highest Cr (VI) removal rate of 14.82 mg Cr^6+^/g VSS/h in this batch reactor. As sulfide could only be produced after Cr (VI) had been used up^18^, it is reasonable to expect the rate of the abiotic reduction of Cr (VI) to be much faster than the rate of biological sulfidogenesis. Therefore, sulfate reduction rate is the rate-limiting step of the two-step indirect removal of Cr (VI) and mainly dictates the removal rate of Cr (VI).

### Microbial community analysis

Approximately, 14324 raw pyrosequencing reads of the 16S rRNA gene were obtained from the sludge samples of the UASB reactor. After quality filtering, approximately 12126 reads with an average read length of 378 bp were used for subsequent analyses. The sequences were clustered into 6484 operational taxonomic units (with ≥97% pairwise sequence identity) for the tested sludge of the UASB reactor (Table S1).

**Figure 3:**
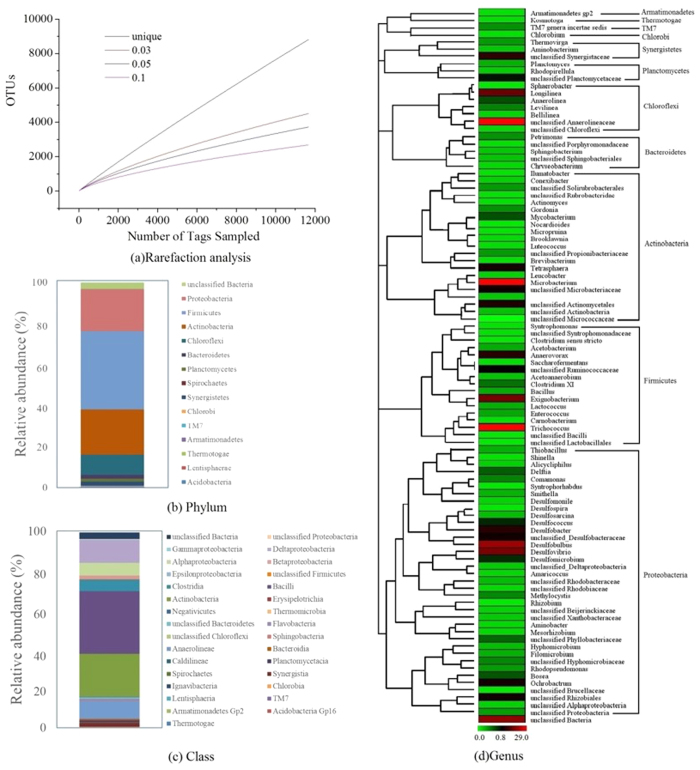
(**a**) Rarefaction analysis of the sludge sample from the UASB reactor. Rarefaction is shown for OTUs that contain unique sequences and OTUs with differences that do not exceed 3%, 5% or 10%. OTUs with ≥97% pairwise sequence identity are assumed to form the same species and genus, respectively; Taxonomic classification of bacterial 16s rRNA gene reads retrieved from UASB reactor at phylum (**b**) and class (**c**) levels using RDP classifier with a confidence threshold of 97%; (**d**) Heatmap showing the relative abundance and phylogenetic relationships of different genera retrieved from the sludge of the UASB reactor. The color indicates the percentage of a genus in total sequences.

Altogether, 15 bacterial phyla were recovered from sludge samples. The majority of 16s rRNA gene sequences belong to Firmicutes representing approximately 38.2% of the total sequences (Fig. 3a). In addition, a significant presence of Proteobacteria (20.6%) and Actinobacteria (22.2%) was also observed. At genus level, *Trichococcus* affiliated to the Bacilli class was dominant in the reactor accounting for 28.1% of the total sequences.

## Discussion

### Fate of sulfate in the sufidogenesis-induced Cr (VI) removal in the UASB reactor

**Figure 4:**
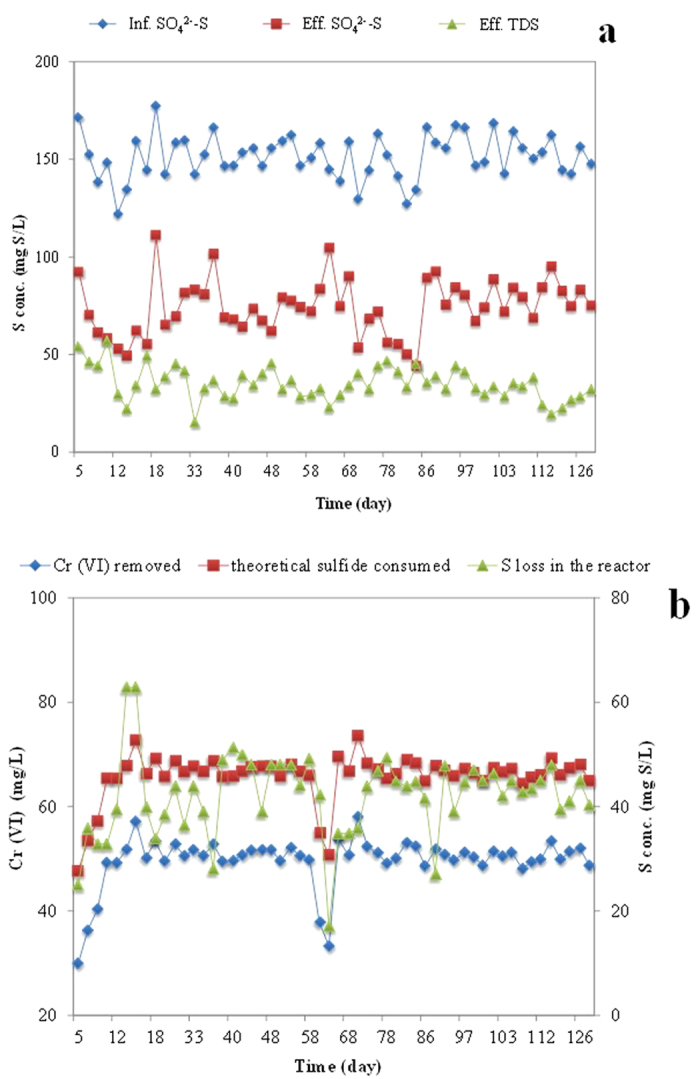
(**a**) Profile of the sulfur compounds of the influent and effluent of UASB reactor; (**b**) Profile of Cr (VI) removal, theoretical sulfide consumption for Cr (VI) removal and the actual sulfur loss during the operation of UASB reactor.

As shown in Eqs 2 and 3, sulfide reduced from sulfate by SRB can be oxidized by chromate into either elemental sulfur or sulfate. The stoichiometry (see Eqs 2 and 3) shows that the mass ratio of oxidized sulfide to reduced chromate is either 0.92 in case sulfide is oxidized to elemental sulfur, or 0.23 if sulfide is oxidized to sulfate. In this study, the average mass ratio calculated from the measured data (see Fig. 4a) was determined to be 0.85, which is very close to the mass ratio of 0.92 when sulfide is oxidized to elemental S. Therefore, sulfide was most possibly oxidized to elemental sulfur instead of sulfate. This is confirmed by the elemental sulfur measurement in the batch test.

**Figure 5:**
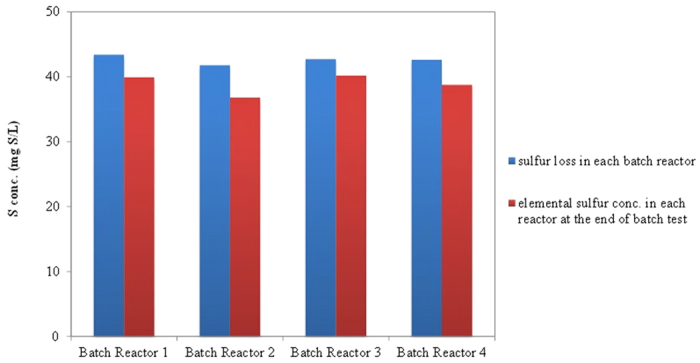
Profile of sulfur loss and elemental sulfur concentrations in each batch reactor at the end of the batch test.

Figure 5 shows the loss of sulfur and resultant elemental sulfur concentration in each batch reactor measured at the end of the batch test. In each batch reactor, about 90–95% of lost sulfur (initial total sulfur [sulfate] concentrations at the beginning of the batch test 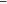 final total sulfur [sulfate + sulfite + thiosulfate + sulfide] concentrations at the end of batch test) was recovered as elemental sulfur. This confirms that in the sulfidogenesis-induced process for the removal of Cr (VI), the sulfide produced from biological sulfate reduction was oxidized to elemental sulfur instead of sulfate.

### Pathways of Cr (VI) removal in UASB under sulfate-reducing conditions

Based on the oxidation of sulfide to elemental sulfur and resultant Cr (VI) removal, the profile of theoretically consumed sulfide (based on the stoichiometric mass ratio of sulfide to Cr^6+^ is 0.92) is shown in Fig. 4b. The profile of time course-based S loss in the reactor (influent total sulfur concentration [sulfate]-effluent total sulfur concentration [sulfate + sulfite + thiosulfate + sulfide]) is also shown in the same figure. As elemental sulfur was not directly measured in this study, the sulfur loss was estimated from the amount of sulfide oxidized to elemental sulfur during reduction of Cr (VI). The ratio of actual sulfur loss (sulfide oxidation to elemental sulfur) to theoretically consumed sulfide in the UASB reactor for the reduction of Cr (VI) was determined to be 0.9 (see Fig. 4b). This indicates that 90% Cr (VI) was removed by sulfide oxidation to elemental sulfur. Hence, about 10% Cr (VI) was removed by ways other than the oxidation of sulfide. Although the intermediary sulfur compounds during biological sulfate reduction (i.e. sulfite and thiosulfate) could also react chemically with Cr (VI), these reactions were reported to be insignificant when compared with those between sulfide and Cr^6+^, especially at pH higher than 6.0^43^. Rather, direct enzymatic chromate reduction (in the presence of organics and Cr (VI)) observed in this study could explain the remaining 10% Cr (VI) removal. This was confirmed by batch test and microbial analysis showing 15% *Microbacterium* (chromate-reducing bacteria) at genus level in the UASB reactor, which contributed 10% Cr (VI) reduction in the UASB reactor.

### Microbial community in UASB reactor for Cr (VI) removal

The dominant classes in the sludge of the UASB reactor were Bacilli, Actinobacteria, and Deltaproteobacteria, accounting for 32.0, 22.2, and 11.7%, respectively (Fig. 3b). In contrast, the bacterial community in the seeding sludge was dominated by Deltaproteobacteria (50.46%), Chlorobi (13.2%), Bacilli (9.5%), and Actinobacteria (9.5%)^44^. The significant changes of the bacterial communities (i.e. from Deltaproteobacteria to Bacilli) suggest that Bacilli might have played an important role in such a Cr (VI)-abundant environment, possibly due to aggregation of bacteria following their acclimation.

Previous studies have shown that *Trichococcus* has the ability to degrade complex organics such as yeast and glucose to simple ones like lactate and acetate^45^. *Trichococcus* might have played a similar role in the UASB reactor in this study particularly because glucose and yeat were two of the carbon sources used. In this way, the simple organics could be further oxidized to inorganic carbon by sulfate or chromate^48^. In the UASB reactor, the SRB groups including *Desulfobulbus*, *Desulfovibrio* and *Desulfobacter*-like species accounted for 11.1% of total sequences (Fig. 3c). These bacteria might be responsible for the sulfidogenic reaction in the reactor. In addition, *Desulfovibrio* was also reported to be able to utilize CrO_4_^2−^ as the alternative electron acceptor besides sulfate^46^. This might have contributed to the loss of Cr (VI) via the direct reduction of Cr (VI). Moreover, the genus *Microbacterium,* which could directly reduce Cr (VI) to Cr (III)^17^ was found to account for 15% of the total sequences (Fig. 3c). The results suggest that the identified and un-classified SRB groups might be responsible for indirect removal of Cr (VI) via sulfidogenic activities in the UASB reactor, while other groups of bacteria including genera *Desulfovibrio, Microbacterium* and other un-classified bacteria might be responsible for the direct reduction of Cr (VI) to Cr (III). The efficient removal of Cr (VI) in the UASB reactor could have resulted from the interactions between different functional groups of bacteria, i.e. the fermenting group (e.g. *Trichococcus*), the SRB group, and the direct chromate-reducing group (e.g. *Microbacterium*).

### Comparison of the biological Cr (VI) removal with other studies

From the engineering perspective, it is more appropriate to express the Cr (VI) removal performance in terms of reduced Cr (VI) concentration per unit time, i.e. mg Cr (VI)/L/h than other ways^47^. In this study, we used a UASB reactor to treat sulfate-laden wastewater mixed with synthetic Cr (VI) in concentration of 50 mg Cr (VI)/L at the HRT of 12 hours. These resulted in 100% removal efficiency, i.e. Cr (VI) removal rate of 4.17 mg Cr (VI)/L/h. During the 4 months of operation, besides the transition period from Phase I to Phase II (the HRT decreased from 24 to 12 hours), the operation of the UASB reactor remained stable. Comparatively, using the tricking filter, the highest Cr (VI) removal rate was 3.03 mg Cr (VI)/L/h in the sequential batch reactor (SBR) mode, and subsequently failed after switching to the continuous mode due to the lost of active biomass^47^. In other batch mode studies, using the mixed cultures under aerobic conditions, the maximum Cr (VI) reduction rates were 2 mg Cr (VI)/L/h and 4.59 mg Cr (VI)/L/h, respectively, with sugar^1^ and molasses^47^ as the carbon source. Using the pure aerobic culture of *Bacillus amyloliquefaciens* as the inoculum, the highest Cr (VI) reduction rate was 2.22 mg Cr (VI)/L/h^49^. And according to the results of our batch test, the maximum rate of Cr (VI) reduction was 6.91 mg Cr (VI)/L/h. Although it is reported that the biological Cr (VI) removal is normally more favorable under aerobic conditions than anoxic/anaerobic conditions^48^,^50^, we still observed a higher anaerobic Cr (VI) removal rate based on the results of the batch mode. So the biological sulfidogenesis-induced Cr (VI) reduction carried out by the SRB (*Desulfobulbus*, *Desulfovibrio* and *Desulfobacter*) together with the direct Cr (VI) reduction carried out by the chromate-reducing bacteria (*Microbacterium*) contributed to the high Cr (VI) removal in the UASB reactor. It is therefore feasible to apply the UASB reactor for the treatment of EW containing large amount of both sulfate and Cr (VI).

Recently, Peng *et al.*^51^ studied the Cr (VI) reduction under biological Fe^3+^ reducing condition. They also proposed the two-pathway model for Cr (VI) removal by calibration and validation, 1) Fe^3+^ reducing to Fe^2+^ and the chemical reaction between Fe^2+^ and Cr^6+^, and 2) direct reduction of Cr (VI) by iron-reducing bacteria. The proportion of each pathway contributing to the reduction of Cr (VI) is closely related to the HRT and organic concentrations in the reactor. As the HRT and organic concentrations are the two factors determining the loading rate in the real application, future work will be conducted on EW treatment in a UASB reactor under different HRT conditions and the organic concentrations to clarify the relationship between the two pathways.

## Methods

### Reactor operation

A lab-scale UASB reactor was set up, with an effective volume of 1.1 L and a diameter of 50 mm (see Fig. S4 in the Supporting Information [SI]). It was initially seeded with sludge taken from a sulfate/sulfite-reducing UASB reactor^52^. The seeding sludge contained a high level of SRB (46% at genus level^44^) with the MLVSS concentration of about 6500 mg/L in the UASB reactor. Synthetic wastewater, whose composition is given in Table S2, contained glucose, yeast, and acetate as the organic carbon sources for the reactor. The stock Cr (VI) solution containing 1000 mg Cr/L was prepared by dissolving 0.2828 mg of 99.5% K_2_Cr_2_O_7_ (analytical grade) into 1 L of ultra-pure water. This was then diluted by 20 times, yielding the influent concentration of 50 mg Cr/L (see Table 3), before feeding it to the UASB via peristaltic pumps. Subsequently, a solution of sodium sulfate of 150 mg S/L was fed to the reactor to induce sulfidogenesis coupled with the reduction of Cr (VI) to Cr (III).

The operating conditions of the UASB reactor are summarized in Table 3. The whole study period was divided into two phases according to the different hydraulic retention times (HRTs), Phase 1 from Day 0 to Day 61 at HRT of 24 h. In order to double the Cr (VI) removal capacity in the UASB reactor, HRT was halved in Phase 2 from Day 64 to Day 128. Correspondingly, the up-flow velocity of the UASB reactor was 0.023 and 0.047 m/h, respectively in each phase (see Table 3). In both phases the influent pH of the reactors were adjusted to 6.5–6.8 (by adding solutions of HCl/NaOH), which corresponded to the effluent pH of 7.7–8.5 (see Fig. 1a). The temperature of the reactor was monitored at 23 ± 1 °C in an air-conditioned room.

### Effect of pH on Cr (VI) removal

The sulfidogenesis-induced removal of Cr (VI) involves two steps, namely the biological sulfate reduction and the abiotic reduction of Cr (VI) by sulfide, both of which is affected by the pH^15^,^53^. Also, the activity of the direct reduction of Cr (VI) depends on the pH^14^,^54^. So pH plays a key role in the removal of Cr (VI) in the UASB reactor, which could be achieved by either sulfidogenesis or by the direct reduction of Cr (VI).

The effect of pH on the removal of Cr (VI) was investigated by batch tests at the end of Phase 2, i.e. on Day 128, with sludge taken from the UASB reactor. Prior to testing, the sludge samples was washed with distilled water in order to remove background substrates (organics, CrO_4_^2−^, SO_4_^2−^, and HS^−^/H_2_S). A total of 20 testing reactors, including 4 batch reactors and 16 control reactors were operated for 48 h (Table 1). Batch Reactors 1 to 4 were used to test the effect of pH on the removal of Cr (VI) at the pH values of 6.0, 7.0, 8.0, and 9.0, respectively in each reactor, which were adjusted by Na_2_HPO_4_/NaH_2_PO_4_ buffer solution.

Besides the abovementioned 4 batch reactors, 16 control reactors were also set up simultaneously in the batch test. Control Reactors 1 to 8 were operated to evaluate possible losses of Cr (VI) through other pathways during the tests, e.g. through chemical reduction/adsorption, or biosorption (see specific operation conditions in Table 1). Non-sludge reactors (Control Reactors 1 to 4, without any inoculum sludge) were operated in parallel to monitor the possible abiotic removal (chemical reduction/adsorption) of Cr (VI); Non-organics/sulfate reactors (Control Reactors 5 to 8, with inoculum sludge and chromate but without sulfate and organics) were operated to correct for the possible losses of Cr (VI) by biosorption. In addition, non-sulfate reactors (Control Reactors 9 to 12, without sulfate but with biomass, organics, and chromate) were operated to test the contribution of the direct reduction of Cr (VI) to Cr (III) to the removal of Cr (VI) by the SRB and other chromate-reducing bacteria in each batch reactor. Non-Cr (VI) reactors (Control Reactors 13 to 16, with COD and sulfate as the only substrates but no Cr (VI)) were operated to examine the effect of Cr (VI) on the sulfidogenesis activity at different pH values ranging 6.0–9.0. Details of testing and operating conditions of the batch and control reactors are given in Table 1. Each of these reactors was a 2-L glass serum flask sealed with a butyl rubber stopper plus aluminium crimp seal. Prior to testing, the reactors were all purged with nitrogen gas (analytical grade) to exclude dissolved oxygen. The temperatures of the reactors were kept at 23 ± 1 °C in an air-conditioned room, and each flask was stirred at 150 rpm by using a magnetic stirrer. All the batch tests lasted for 48 hours.

### Chemical/physical analysis

During the operation of the UASB reactor, both the influent and effluent were sampled regularly for chemical/physical analysis. In the batch tests, mixed liquor samples were taken from each reactor by using a 10-mL syringe. The samples were immediately filtered through disposable Millipore filters (0.45 um pore size). Total dissolved Cr (VI) concentration was determined by colorimetric method according to the Standard Method^55^. Total dissolved chromium concentration was measured using an atomic absorption spectrophotometer (AAS). Since Cr (VI) and Cr (III) are the predominant forms of Cr in water solution^56^, Cr (III) is usually estimated as the difference between the total chromium and hexavalent chromium^57^. Sulfide was preserved by NaOH and ZnAc according to the Standard Method and then measured immediately by the methylene blue method in order to prevent dissolved sulfide loss from volatilization and abiotic oxidation^55^. Sulfate, thiosulfate and acetate were analyzed with an ion chromatograph (HIC-20A super) equipped with a conductivity detector and an IC-SA2 analytical column. The elemental sulfur in the four batch reactors was extracted by tetrachloroethylene prior to its characterization using LC-20A high-performance liquid chromatography (Shimadzu, Japan)^58^. Sulfite was measured by the titration method^55^. COD was measured by Hach Tech method. Sludge samples of the UASB reactor were taken from the bottom, middle, and top of the reactor before measuring the mixed liquor suspended solid (MLSS)/mixed liquor volatile suspended solid (MLVSS) concentrations, and the extracellular polymeric substances (EPS). MLSS/MLVSS were measured according to the Standard Method. The EPS were first extracted from sludge using a formaldehyde-NaOH method^59^. The carbohydrate or polysaccharides (PS) contents in the EPS were then quantified using the phenol-sulfuric acid method with glucose as the standard^60^. The protein (PN) content of the EPS was further determined from a modified Lowry colorimetric method (DC Protein Assay, BioRad) with bovine albumin serum as standard^61^. The pH and temperature were monitored using a multi-meter electrode during each test (WTW multi 3420). The results reported are average values of three replicates. Standard deviation values were under 5%.

### Microbial analysis

Sludge samples from the UASB reactor were collected at the end of Phase 2 (Day 128) for analysis of the bacterial diversity. The DNA extraction, PCR reactions and thermocycles, the preparation of library for 454 pyrosequencing, and the data analysis was referred to Jiang *et al.*^62^. Briefly, the bacterial 16S rRNA gene was amplified with barcode sequences primer pair 515F and 926R (Table S3)^63^. The PCR products were then purified using the TaKaRa Agarose Gel DNA Purification Kit (TaKaRa, China) and quantified before sequencing on the ROCHE 454 FLX Titanium platform (Roche, Basel, Switzerland). The raw reads were processed using the pyrosequencing pipeline initial process of the Ribosomal Database Project (RDP)^64^ for quality filtering and noise removal^65^. The remained sequences were then aligned and clustered into operational taxonomic units (OTUs) using the software MOTHUR ver. 1.17.0^66^. The taxonomic classifications were assigned using the RDP Classifier^67^. Statistical parameters including the rarefaction curves and diversity indices (ACE and Chao1) were generated in MOTHUR. Good’s coverage was calculated according to GOOD^68^.

## Additional Information

**How to cite this article**: Qian, J. *et al.* An Exploratory Study on the Pathways of Cr (VI) Reduction in Sulfate-reducing Up-flow Anaerobic Sludge Bed (UASB) Reactor. *Sci. Rep.* **6**, 23694; doi: 10.1038/srep23694 (2016).

## Supplementary Material

Supplementary Information

## Figures and Tables

**Table 1:** Experimental conditions for all the batch reactors and control reactors in the batch test.

	pH	COD conc. (mg/L)	SO_4_^2−^ conc. (mg S/L)	Cr (VI) conc. (mg/L)	Biomass conc. (mg VSS/L)
Batch Reactor 1	6.0	150	75	50	488
Batch Reactor 2	7.0	150	75	50	472
Batch Reactor 3	8.0	150	75	50	466
Batch Reactor 4	9.0	150	75	50	484
Non-sludge reactors					
Control Reactor 1	6.0	150	75	50	–
Control Reactor 2	7.0	150	75	50	–
Control Reactor 3	8.0	150	75	50	–
Control Reactor 4	9.0	150	75	50	–
Non-COD/sulfate reactors					
Control Reactor 5	6.0	–	–	50	459
Control Reactor 6	7.0	–	–	50	441
Control Reactor 7	8.0	–	–	50	466
Control Reactor 8	9.0	–	–	50	447
Non-sulfate reactors					
Control Reactor 9	6.0	150	–	50	494
Control Reactor 10	7.0	150	–	50	508
Control Reactor 11	8.0	150	–	50	482
Control Reactor 12	9.0	150	–	50	498
Non-Cr (VI) reactors					
Control Reactor 13	6.0	150	75	–	462
Control Reactor 14	7.0	150	75	–	443
Control Reactor 15	8.0	150	75	–	452
Control Reactor 16	8.0	150	75	–	458

**Table 2:** Kinetic results of the batch test of the pH influence on Cr (VI) reduction, including four batch reactors (Batch Reactor 1 to 4) and four control reactors (Control Reactor 13 to 16).

	pH	Biomass-specific SO_4_^2−^ reduction rate (mg S/g VSS/h)	Biomass-specific Cr (VI) reduction rate (mg Cr^6+^/g VSS/h)
Batch Reactor 1	6.0	4.85	5.42
Batch Reactor 2	7.0	10.84	11.58
Batch Reactor 3	8.0	14.07	14.82
Batch Reactor 4	9.0	8.80	6.67
Non-Cr (VI) reactors			
Control Reactor 13	6.0	9.19	–
Control Reactor 14	7.0	15.17	–
Control Reactor 15	8.0	19.80	–
Control Reactor 16	9.0	11.09	–

**Table 3:** Conditions of UASB reactor for Cr (VI) removal.

	Phase I	Phase II
Average Inf. COD (mg/L)	210	
Average Inf. Cr^6+^ (mg/L)	50	
Average Inf. SO_4_^2−^-S (mg/L)	150	
Influent pH	6.5~6.8	
HRT (h)	24	12
Up-flow velocity (m/h)	0.023	0.047
