# Supplementary material for: An Exploratory Study on the Pathways of Cr (VI) Reduction in Sulfate-reducing Up-flow Anaerobic Sludge Bed (UASB) Reactor
Source: Sci Rep. 2016 Mar 29;6:23694. doi: 10.1038/srep23694 (PMC4810426; doi:10.1038/srep23694)
Supplement: Supplementary Information [file srep23694-s1.pdf]

Supporting Information

An Exploratory Study on the Pathways of Cr (VI) Reduction in Sulfate-reducing Up-flow Anaerobic Sludge Bed (UASB) Reactor

Jin Qian[1,2], Li Wei[2,5], Rulong Liu[2], Feng Jiang[6], Xiaodi Hao[7], Guang-Hao Chen[2-5]*

[1]School of Natural and Applied Sciences, Northwestern Polytechnical University, Xi'an, China

[2]Department of Civil and Environmental Engineering, The Hong Kong University of Science and Technology, Clear Water Bay, Kowloon, Hong Kong, China

[3] Water Technology Center, The Hong Kong University of Science and Technology, Hong Kong, China

[4] Hong Kong Branch of Chinese National Engineering Research Center for Control & Treatment of Heavy Metal Pollution, The Hong Kong University of Science and Technology, Hong Kong, China

[5] Wastewater Treatment Laboratory, FYT Graduate School, The Hong Kong University of Science and Technology, Nansha, Guangzhou, China

[6]School of Chemistry & Environment, South China Normal University, Guangzhou, China

[7]Beijing University of Civil Engineering and Architecture, Beijing, China

Correspondence and requests for materials should be addressed to

G.H. Chen (email: *ceghchen@ust.hk)*

Table S1 Similarity-based OTUs and species richness estimates with a confidence threshold of 97%

OTUs	ace	chao	shannon	simpson	coverage
6484	4788.178	3348.065	5.282166	0.037568	84.66%

Table S2. Composition of stock and trace element solutions.

Stock solution			Trace element solution	
Component		Concentration (g/L)	Component	Concentration (g/L)
Organic component	Glucose	19.57	$FeCl_3 \cdot 6H_2O$	2
	Yeast extract	26.1	H_3BO_3	0.2
	Sodium acetate	9.786	$CuSO_4$	0.05
Inorganic component	NH_4Cl	18.45	KI	0.08
	K_2HPO_4	1.92	$MnSO \cdot 4H_2O$	0.25
	KH_2PO_4	0.72	$ZnSO_4 \cdot 7H_2O$	0.15
	$MgCl_2 \cdot 6H2O$	8.32	$CoCl_2 \cdot 6H_2O$	0.2
	$CaCl_2$	5.2		
	$NaHCO_3$	62.4		

Table S3 Primer of the DNA amplification for UASB reactor

Barcode Sequence	Primer
ATGCTACGTC	(V1-V3）<br>8F: 5’-AGAGTTTGATCCTGGCTCAG-3’<br>533R: 5’-TTACCGCGGCTGCTGGCAC-3’

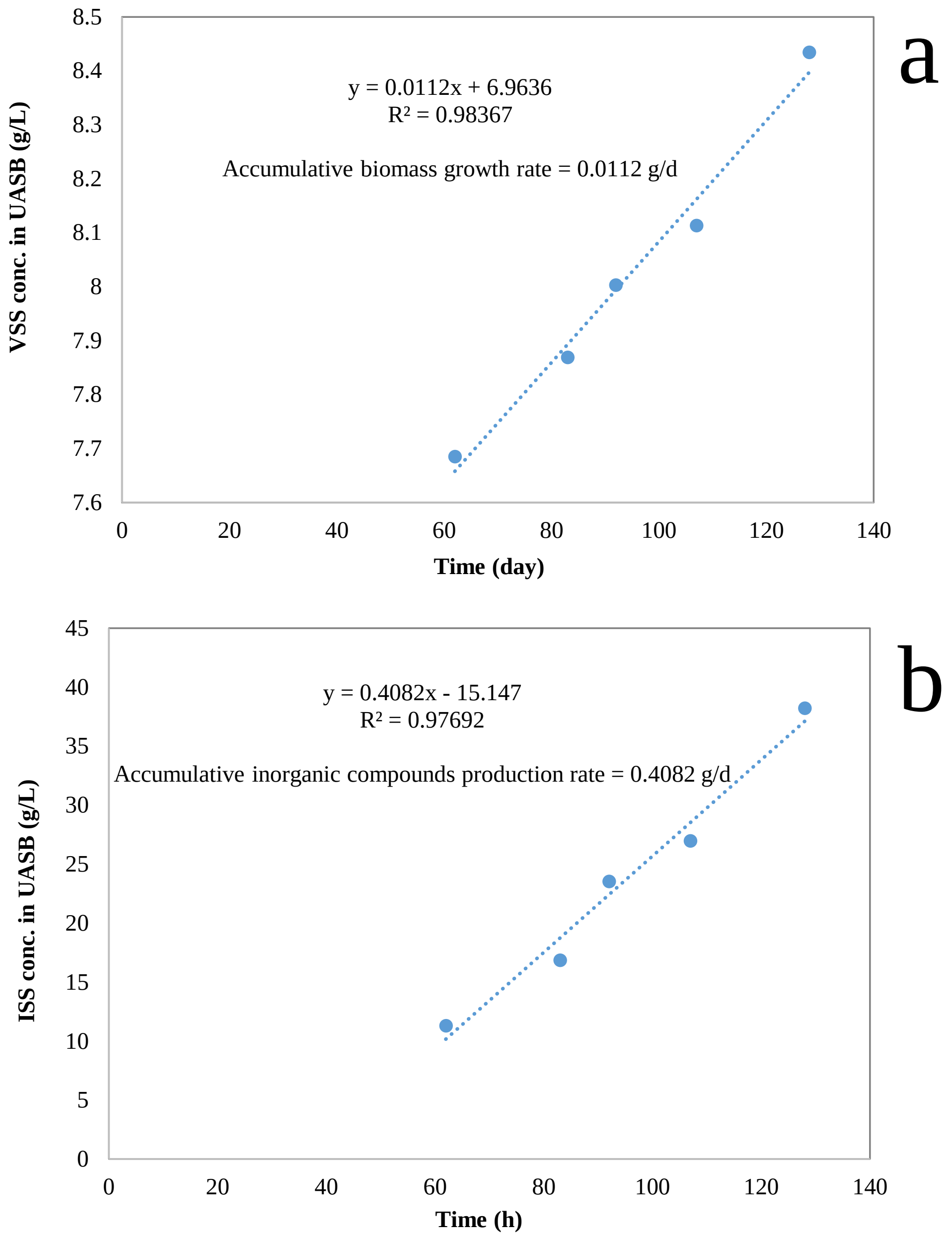

Fig. S1 Sludge production during the UASB operation:

(a) accumulative biological sludge (expressed as VSS, including biological sludge washing out and sampling from the reactor) production**,** when HRT = 12 h, biological sludge production in UASB was calculated as accumulative biomass growth rate (g VSS/d) ÷ average COD removal rate (average Inf. COD* × average COD removal efficiency** × flow rate***, g COD/d).

*average Inf. COD = 210 mg/L at HRT_{SRUSB} = 12 h;

**average COD removal efficiency = 92.5%;

***flow rate = 2.2 L/d in Phase 3 at HRT_{SRUSB} = 3 h.

So the specific biological sludge production rate is 0.026 g VSS/g COD

(b). chemical sludge (expressed as ISS, including chemical sludge washing out and sampling and withdrawn from the reactor) production

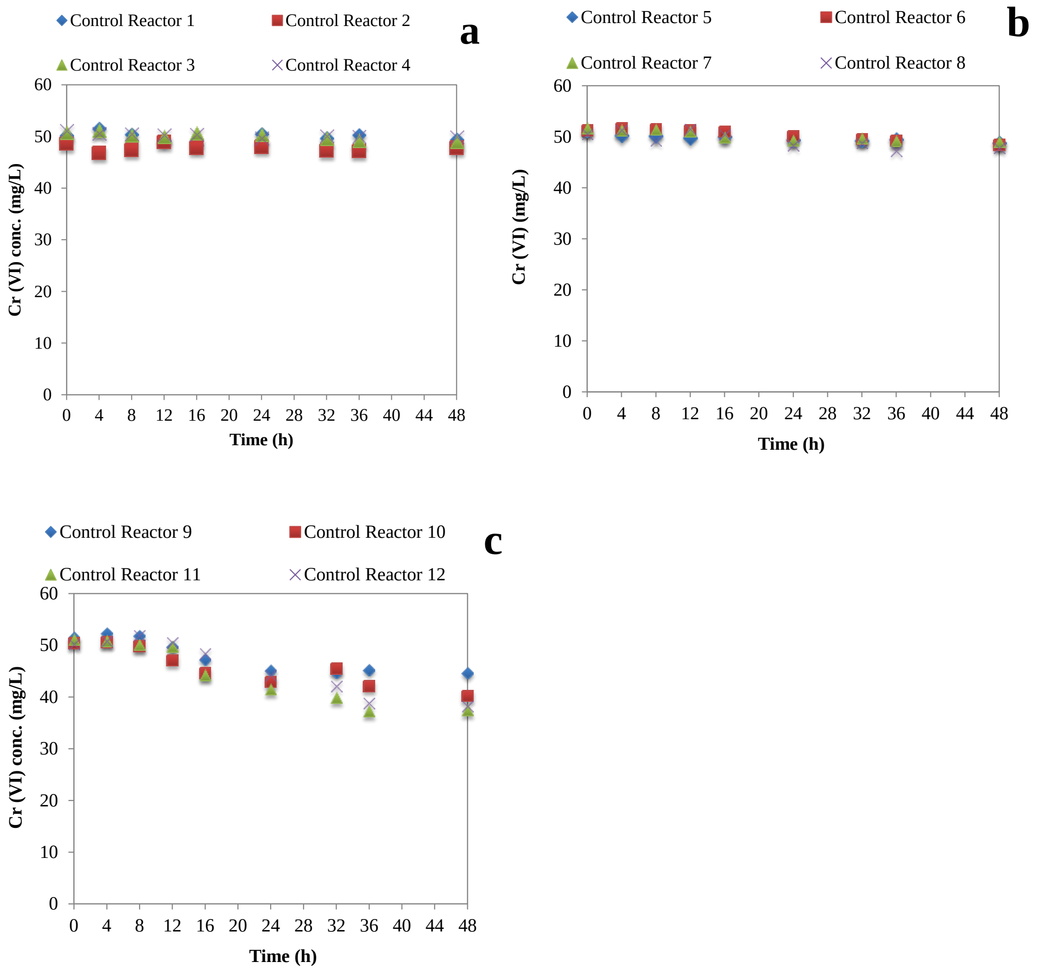

Fig. S2 Cr (VI) profile of Control Reactor 1 to 4 **(a)**, Control Reactor 5 to 8 **(b)** and Control Reactor 8 to 12 **(c)**.

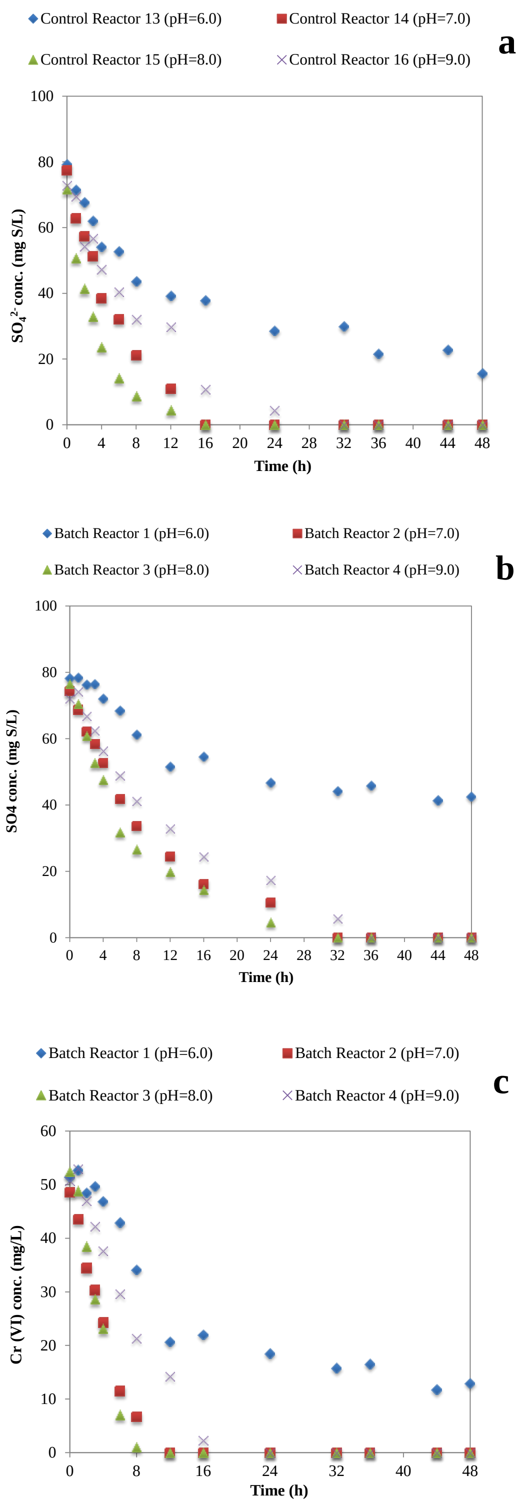

Fig. S3 Sulfate profile (SO_4^{2-} concentration versus time) in **(a)** Control Reactor 13 to 16, without Cr (VI) dosed and **(b)** Batch Reactors 1 to 4, with Cr (VI) dosed under each pH condition; **(c)** Cr (VI) profile (Cr (VI) concentration versus time) of Batch Reactors 1 to 4.

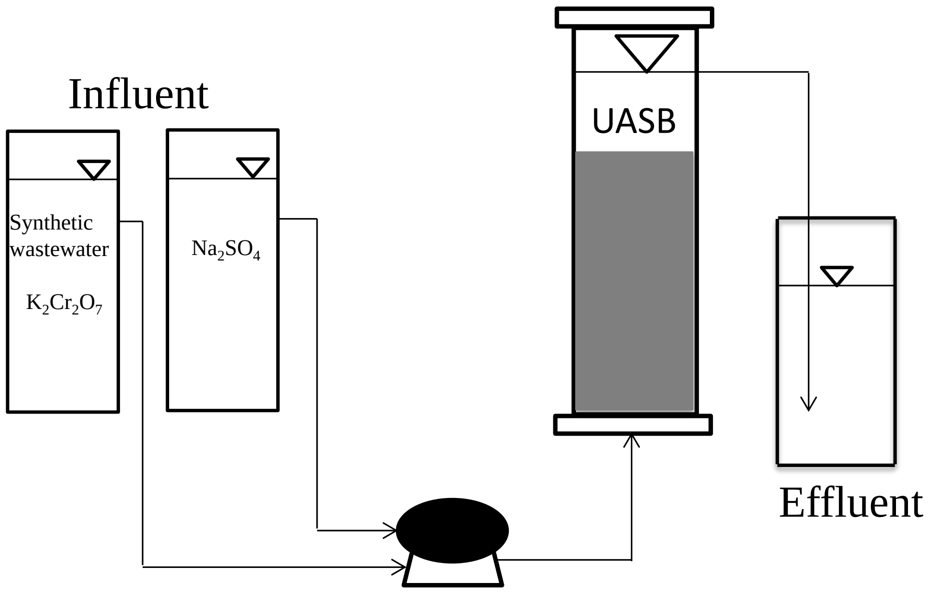

Fig. S4 Schematic of the UASB reactor for Cr (VI) removal through sulfidogenic activity
